# Risk factors of laryngeal cryptococcosis: A case report

**DOI:** 10.1016/j.mmcr.2019.04.009

**Published:** 2019-04-27

**Authors:** Orlando Quintero, Polina Trachuk, Michael Z. Lerner, Judy Sarungbam, Liise-anne Pirofski, Sun O. Park

**Affiliations:** a Division of Infectious Diseases, Department of Medicine, Albert Einstein College of Medicine and Montefiore Medical Center, Bronx, NY, USA; b Department of Otolaryngology, Albert Einstein College of Medicine and Montefiore Medical Center, Bronx, NY, USA; c Department of Pathology, Albert Einstein College of Medicine and Montefiore Medical Center, Bronx, NY, USA

**Keywords:** Cryptococcosis, Larynx, Endobronchial, Corticosteroid, *Mycobacterium avium-Intracellulare* (MAI), Mannose-binding lectin deficiency

## Abstract

Cryptococcal infections are acquired by inhalation of encapsulated yeast cells or basidiospores. While *Cryptococcus* has a propensity to invade the lungs and central nervous system, other sites can be affected. Laryngeal cryptococcosis is rare with less than 30 previously reported cases, which commonly occurred in apparently immunocompetent hosts on inhaled corticosteroids. We present a case of laryngeal cryptococcosis with a long-term inhaled corticosteroid use, co-infection of pulmonary *Mycobacterium avium-intracellulare*, and mannose-binding lectin deficiency.

## 1 Introduction

Cryptococcal disease is rare, while infection is common [1]. Cryptococcus can cause asymptomatic colonization in exposed individuals [2]. The great majority of cases of cryptococcal disease occur in patients with underlying cellular immune deficiency, most commonly HIV/AIDS [1]. While cellular immune deficiency is a major risk factor for cryptococcosis, B cell defect, mannose-binding lectin (MBL) or Fc-γ receptors (FCGR) polymorphisms may predispose patients to cryptococcosis [1,3]. Since laryngeal cryptococcosis was first described in an immunocompetent host in 1975, inhaled corticosteroid use has come to light as a risk factor for cryptococcal disease in apparently immunocompetent hosts [4]. In this report, we describe a case of laryngeal cryptococcosis and his plausible risk factors of laryngeal cryptococcosis including inhaled corticosteroid use, *Mycobacterium avium-intracellulare* (MAI) infection, and MBL deficiency, and discuss key points of laryngeal cryptococcosis from previous case reports.

## 2 Case

An 80-year-old Puerto Rican man with asthma and chronic obstructive pulmonary disease (COPD) was referred to our medical center's otolaryngology office for 2 months (day −60) of hoarseness and sensation of a lump in the throat. He didn't have fever, weight loss, night sweats, or neurological symptoms (day 0). He had chronic cough productive of yellowish sputum and dyspnea requiring a fluticasone-salmeterol (500/50 mcg) one puff twice daily over 10 years and intermittent COPD exacerbations requiring a short course of oral prednisone every 2–3 months for a couple of years (day −730 to 0). The patient quit smoking 40 years ago after a 20-pack-year of cigarette smoking. He did not have a history of any serious infections as a child.

**Fig. 1:**
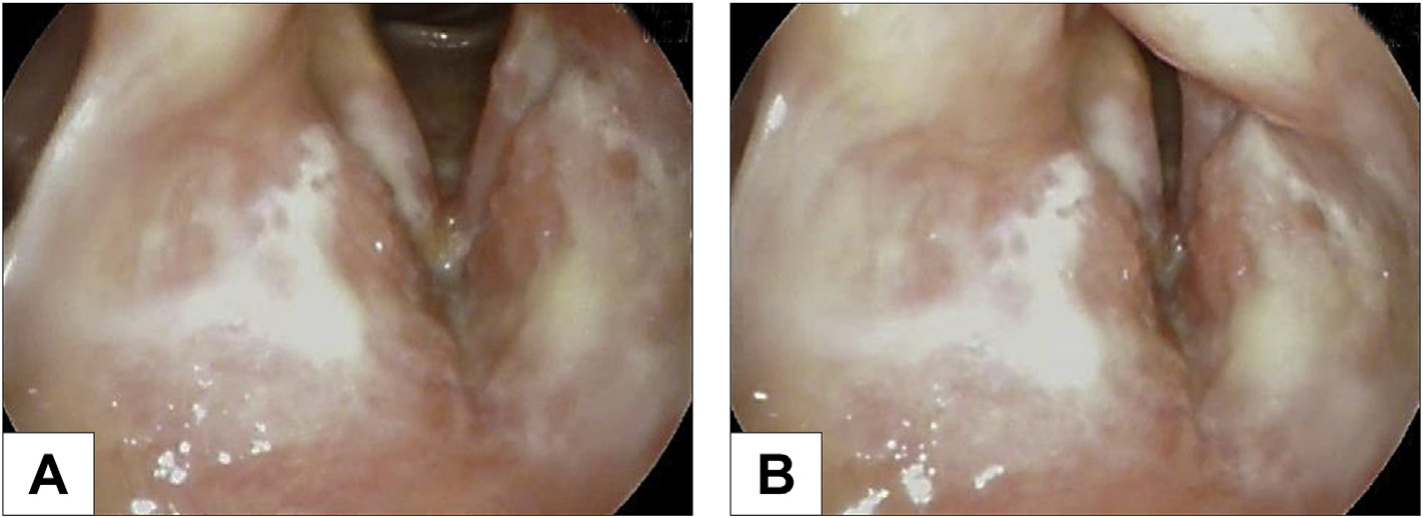
Laryngoscopy at presentation showed erythema and edema of the vocal folds, whitish mucopurulent material coating the endolarynx, and mucosal irregularity of the vestibular folds extending upward along the laryngeal surface of the epiglottis during vocal folds abduction (A) and adduction (B).

**Fig. 2:**
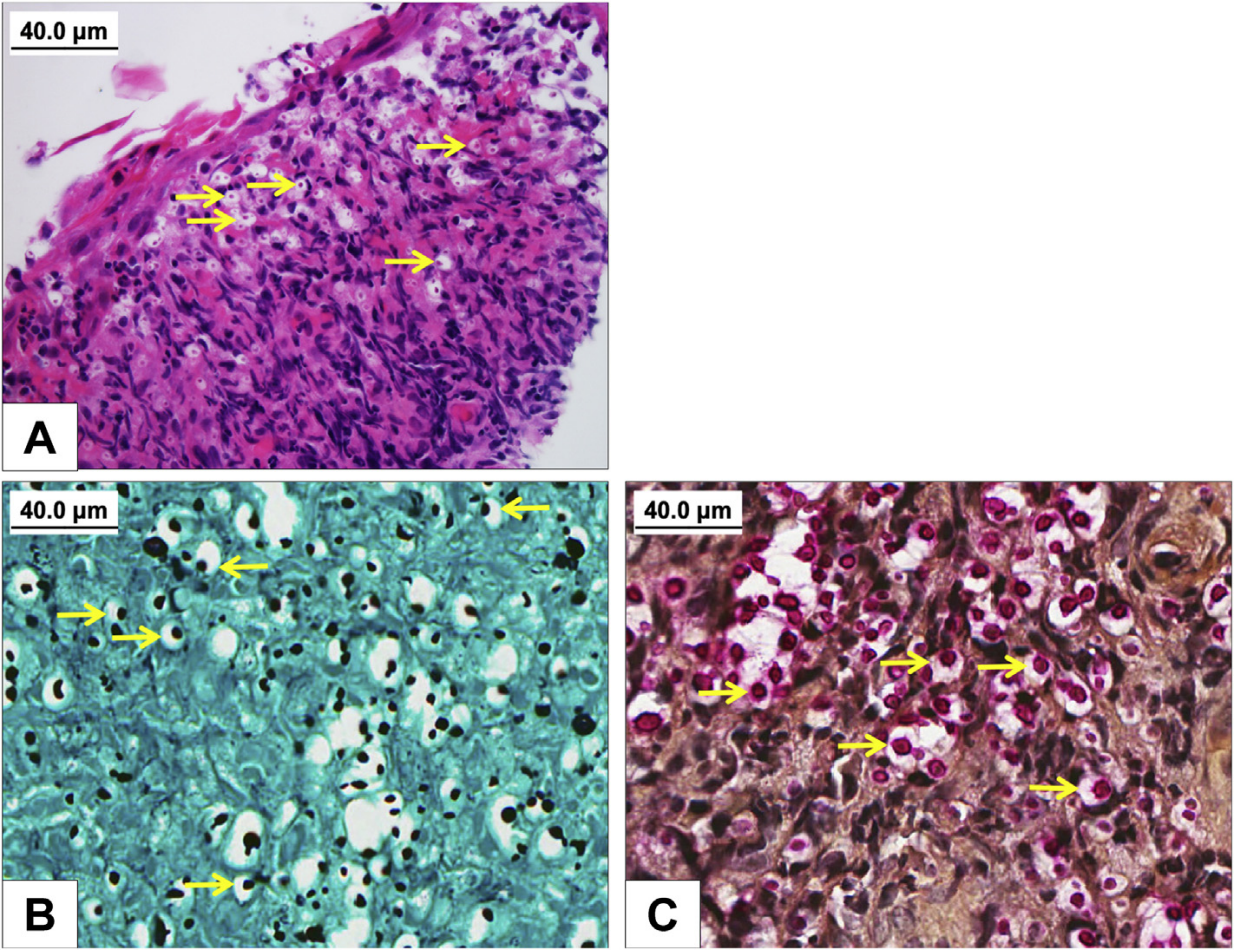
Hematoxylin and eosin stained section shows an ulcerated squamous mucosa, with inflamed granulation tissue and admixed numerous encapsulated yeast form fungal organisms, (A) which are positive for Grocott-Gomori methenamine-silver (B) and mucicarmine (C) stains, consistent with *Cryptococcus Spp*. Arrows indicate encapsulated yeast form fungal organisms.

The patient was noted to be well-appearing with unremarkable physical examination other than mild scattered crackles and wheezes on lung auscultation (day 0). A flexible laryngoscopy and stroboscopy, however, revealed erythema and edema of the vocal folds, whitish mucopurulent material coating the endolarynx, and mucosal irregularity and ulceration of the vestibular folds extending upward along the laryngeal surface of the epiglottis (day 0) (Fig. 1). Empiric 1–2 week courses of oral clotrimazole and nystatin for clinically suspected laryngeal candidiasis, and amoxicillin-clavulanate did not alleviate his symptoms or improve his laryngeal findings. Thereafter, microbiology testing and tissue biopsy were pursued (day +49). Bacterial, acid-fast bacilli (AFB), and fungal cultures of whitish mucus did not yield any organisms. Histopathologic assessment revealed three small fragments of biopsied vestibular folds lesions showing squamous mucosa with ulceration and inflamed granulation tissue, admixed with variably sized round encapsulated yeast forms fungal organisms. These yeast forms were positive for hematoxylin and eosin, Grocott-Gomori methenamine-silver (GMS) and mucicarmine stains, consistent with *Cryptococcus* species (Fig. 2). Immunohistochemical stain, and molecular test confirmed a diagnosis of *Cryptococcus neoformans*. Cryptococcal antigen titer in the serum was 1:20 (day +70).

The patient recalled cleaning up massive pigeon droppings on his terrace 3 months prior to the onset of his symptoms (day −150). His initial white blood cell (WBC) count was 13.1 × 10^9^/L (neutrophil 8.9; lymphocyte 2.8; monocyte 1.0) (day 89). Blood for AFB and fungal cultures were negative (day +92). Cerebrospinal fluid analysis revealed no evidence of meningitis (day +92). Comparing prior CT scans over 3 years, CT of the chest showed new thickening and narrowing of the distal trachea with extensive secretions and impaction of multiple bilateral lower lobe branch bronchi and stable mild bronchiectasis and a few micronodules (day +93). Subsequent bronchoscopy demonstrated a nodular lesion in the narrowed distal trachea, which was biopsied (day +97). Histopathologic exam of the biopsied samples showed tracheal mucosa with focal ulceration, inflammation, fibrosis and rare yeast forms, compatible with *Cryptococcus* species. Multiple respiratory cultures obtained from the bronchoscopy and expectorated sputum specimens were positive for MAI, but *Cryptococcus* was not detected (day +97). An immunodeficiency work up was performed (day +70 to +133), including HIV antigen/antibody, lymphocyte subsets (T-cells, B-cells, natural killer cells), immunoglobulin levels, complement levels, neutrophil oxidative burst assay, MBL level, anti-granulocyte-macrophage colony-stimulating factor (anti-GM CSF) autoantibodies, and Mendelian susceptibility to Mycobacterial disease panel: all were unremarkable, but MBL level was significantly low at 6 ng/ml.

The patient was placed on fluconazole 400mg daily and a salmeterol inhaler instead of a fluticasone-salmeterol inhaler to avoid inhaled corticosteroid (day +91). Three months after fluconazole initiation (day +181), the patient's hoarseness resolved with near complete resolution of laryngeal findings. At five months after fluconazole initiation (day +241), his laryngoscope exam had completely normalized. Due to persistent cough productive of thick respiratory secretions and repeatedly positive MAI from respiratory cultures, the patient was started on azithromycin, rifampin and ethambutol to target MAI at 3–4 months after fluconazole initiation (day +190). Fluconazole trough level was measured due to interaction between fluconazole and rifampin, which was well above the general minimal inhibitory concentration (day +300). A follow-up CT of chest revealed a new occlusion of right middle lobe bronchus and persistent tracheal stenosis, for which the patient underwent balloon dilatation and cryotherapy in the distal trachea (day +239). Eventually, a tracheal stent was placed for non-resolving tracheal stenosis resulting in improvement of tracheal stenosis (day +342). Serum cryptococcal antigen became undetectable after 10–11 months of fluconazole therapy (day +401). The patient completed a total 12 months of fluconazole therapy (day +456) and is planned for 12 months of MAI therapy after sputum AFB conversion.

## 3 Discussion

Cryptococcal disease or cryptococcosis is an invasive fungal infection caused by *Cryptococcus* species. Most human disease is caused by two species, *Cryptococcus neoformans and Cryptococcus gattii* among more than 30 known *Cryptococcus* species [2,5]. About 95% of cryptococcal infections are caused by *C. neoformans* serotype A with remaining by *C. neoformans* serotype D and *C. gattii* [5]. *C. neoformans* is found worldwide from droppings of birds, especially pigeons, turkeys, and chickens and rotting vegetation or wood of certain trees such as eucalyptus and coniferous trees [2,5]. *C. gattii* was thought to be restricted to Australia and other tropical and subtropical regions associated with exposure to eucalyptus species. However, outbreaks of *C. gattii* in Pacific Northwest of North America within the past two decades expanded epidemiological knowledge, linking various trees specific to temperate regions as additional sources of *C. gattii* infection [5]. Risk factors for cryptococcosis include HIV infection, organ transplantation, use of steroid or immunosuppressive agents, malignancy, sarcoidosis, idiopathic CD4^+^ lymphopenia, rheumatologic diseases, and anti-GM CSF autoantibodies [5]. Different from *C. neoforman*s that causes infection more commonly in immunocompromised hosts, most *C. gattii* infections were found in apparently immunocompetent hosts [5]. However, apparently immunocompetent hosts with cryptococcosis may have subclinical immune defects associated with B cell defects or genetic defects [1,3,5]. Furthermore, a study of population comparison showed that *C. gattii* patients are more likely to be immunosuppressed than the compared population [6].

Laryngeal cryptococcosis is a rare disease, which might be caused by hematogenous spread from lungs or direct deposit by inhaled aerosolized organisms [7]. To our knowledge, less than 30 cases of laryngeal cryptococcosis have been reported [4,8,9] since first described in 1975 [10]. Among cases in which species of *Cryptococcus* was reported, only one case was caused by *C. gattii* [7], and the rest were by *C. neoformans* [4,8,9]. The most recent case reports review of laryngeal cryptococcosis by Wong et al. points towards inhaled corticosteroid use, particularly high dose as the most significant risk factor with 45% (10/22) of corticosteroid use by cases [4]. They reported that only 32% (7/22) of cases had a significant systemic immunocompromised status with HIV or systemic corticosteroids exposure [4]. Inhaled corticosteroids as anti-inflammatory agents improve symptoms and decrease frequency of exacerbations of COPD by reducing recruitment and activation of inflammatory cells in the airway [11]. Despite their benefit, inhaled corticosteroids may also cause local injury by inducing direct toxic effects on the airway epithelium, increasing epithelial apoptosis, and impairing repair and immune response to pathogens [11].

Except for the patients who were lost to follow-up, all patients including four HIV patients were cured of laryngeal cryptococcosis [4]. All patients had hoarseness or dysphonia [4,8,9]. Rarity of disease, variable nonspecific laryngeal endoscopic findings, and need for tissue biopsy are challenging conditions to make a correct diagnosis [9]. Malignancy and fungal infections such as *Cryptococcus, Histoplasma, Blastomyces, Coccidioides,* and *Paracoccidiodes* species may be considered when the patients are refractory to empiric local anti-fungal agents or short courses of low dose of fluconazole against suspected *Candida*. While the latest Infectious Diseases Society of America recommends 6–12 months of fluconazole 400mg daily for patients without central nervous system disease, fungemia, or immunocompromised status, laryngeal cryptococcosis was not specifically addressed [12]. Most laryngeal cryptococcosis patients were cured with 4 weeks to 1 year of anti-fungal agent alone, of which fluconazole 400mg daily was used most commonly with at least 6 weeks of duration [4,9]. Inhaled corticosteroid was discontinued or reduced in a half of users while reports of the rest of users (a half) are not available [4]. A couple of patients recovered with surgical excision alone [4], and a couple of patients with anti-fungal laser therapy in addition to fluconazole [13]. Subglottic involvement may require longer treatment [9].

Only three laryngeal cryptococcosis cases including ours also had a tracheobronchial involvement [10,13]. Endobronchial cryptococcosis, a cryptococcal infection of tracheobronchial tree is another rare entity. According to the latest case reports review, there are less than 25 reported cases of endobronchial cryptococcosis, and the vast majority (19/21 cases) of endobronchial cryptococcosis cases had lung or mediastinum involvement [14].

Cryptococcosis can occur with other pathogens such as tuberculosis and nocardiosis [2]. A study of co-infection of *C. neoformans* and MAI in acquired immune deficiency syndrome (AIDS) patients showed a high correlation between *C. neoformans* and disseminated MAI infections [15]. Authors speculated that co-infection of *C. neoformans* and MAI might be due to exposure to their common habitats in bird droppings [15]. An *in vitro* study suggested that macrophages pre-infected with MAI were markedly impaired for killing of intracellular *C. neoformans* in cultured human monocyte-macrophages secondary to a reduction of superoxide production [16]. To our knowledge, this is the first case report of laryngeal cryptococcosis in a patient with pulmonary MAI.

Mannose-binding lectin (MBL) plays an important role in the innate immune response, defending against invading microorganisms by activation of lectin-complement pathway [1,17,18]. MBL deficiency is quite common, affecting 5–30% of people worldwide [19]. Different from severe MBL deficiency in children or immunocompromised hosts which has been associated with increased risk of infections, the clinical impact of MBL deficiency in adults remain controversial [19]. A German study showed that severe MBL deficiency (MBL ≤ 50 ng/ml) without any other immunodeficiency was significantly more frequent in patients with severe and/or recurrent infections. Similarly, a British study showed a higher prevalence of severe MBL deficiency (MBL < 75 ng/ml) in the patients with recurrent infection [19]. A study by Ou et al. showed that mannose-binding lectin (MBL) deficiency caused by *MBL2* gene polymorphisms was significantly associated with cryptococcal meningitis in HIV-negative Chinese patients, particularly in immunocompetent patients [18]. However, a small study of HIV-negative Australian patients didn't demonstrate association between MBL deficiency and cryptococcosis [20]. While further studies between MBL deficiency and cryptococcosis are yet to come, we speculate that severe MBL deficiency in our patient might have contributed to laryngeal cryptococcosis.

It is obvious that only a fraction of apparently immunocompetent hosts who used inhaled corticosteroids for many years and exposed to a ubiquitous environmental fungus, *C. neoformans* developed laryngeal cryptococcosis. In this respect, our case highlights that a possibility of a subtle immune deficiency may be further investigated when clinicians encounter cryptococcal disease in apparently immunocompetent hosts. This knowledge may guide to appropriate treatments and precautions against infections in the future. Furthermore, co-infection of *Cryptococcus* and MAI might be associated with interactions between these two microorganisms and the host's impaired immune system. Our case features MAI infection and MBL deficiency in addition to inhaled corticosteroid use as potential risk factors for laryngeal cryptococcosis.

## Conflict of interest

The authors have no conflicts of interest to declare.
